# Supplementary material for: Comparative Pre-Clinical Analysis of CD20-Specific CAR T Cells Encompassing 1F5-, Leu16-, and 2F2-Based Antigen-Recognition Moieties
Source: Int J Mol Sci. 2023 Feb 12;24(4):3698. doi: 10.3390/ijms24043698 (PMC9966244; doi:10.3390/ijms24043698)
Supplement: Supplementary file 1 [file ijms-24-03698-s001.zip › ijms-1923854-supplementary.pdf]

Supplementary Materials:

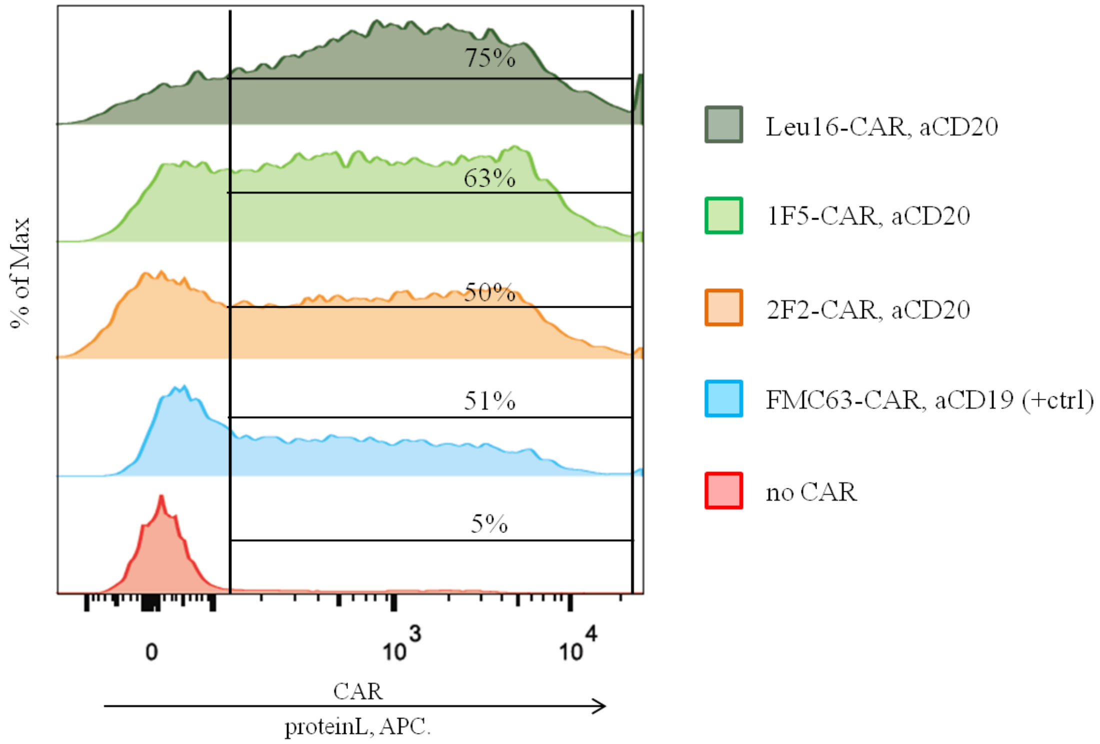

Figure S1. Similar surface expression of all the CARs tested was achieved following transduction of primary human T cells, as assayed by proteinL staining and flow cytometry

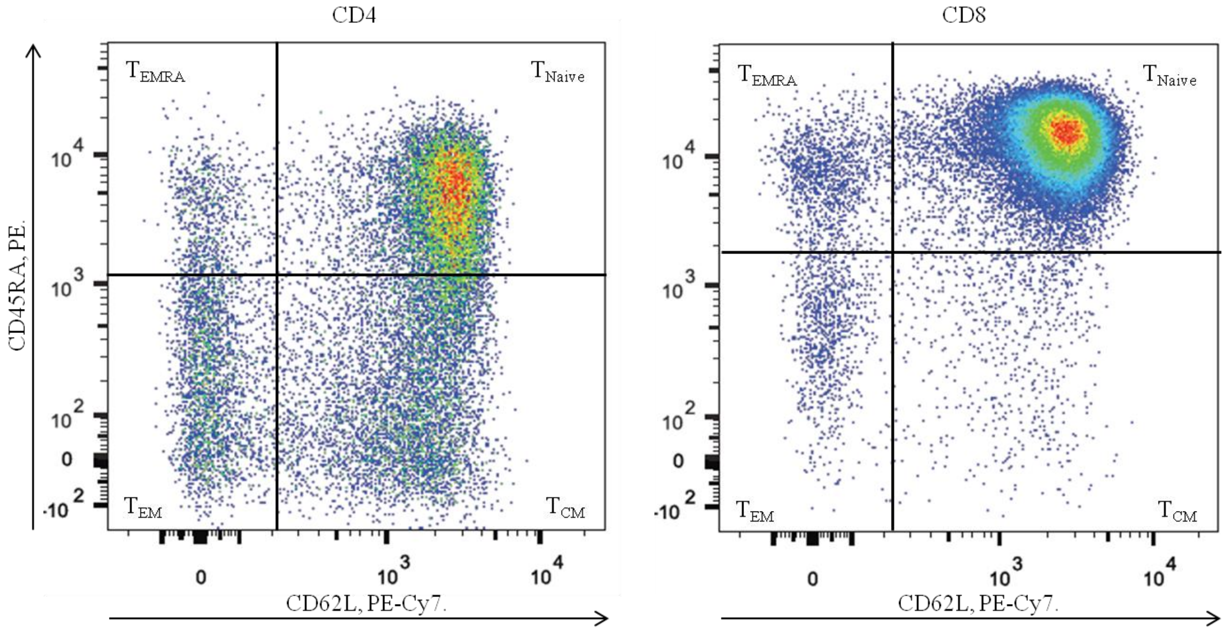

Figure S2. Strategy of TN, TCM, TEM and TEMRA cell subpopulations gating among CD4+ and CD8+ CAR T cells on day 21 after isolation.

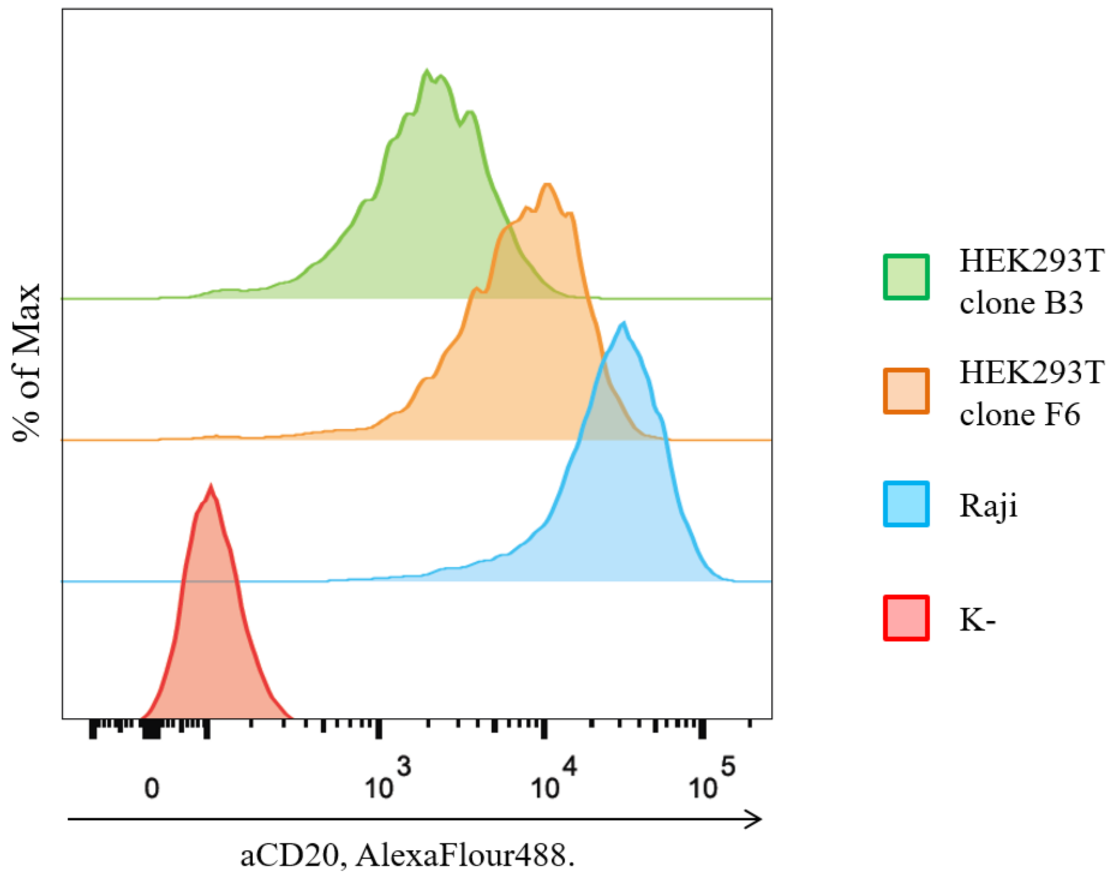

Figure S3. Monoclones of HEK293T-CD20 show lower CD20 expression compared to the CD19+ CD20+ lymphoma Raji cell line, as assayed by anti-CD20 staining and flow cytometry.

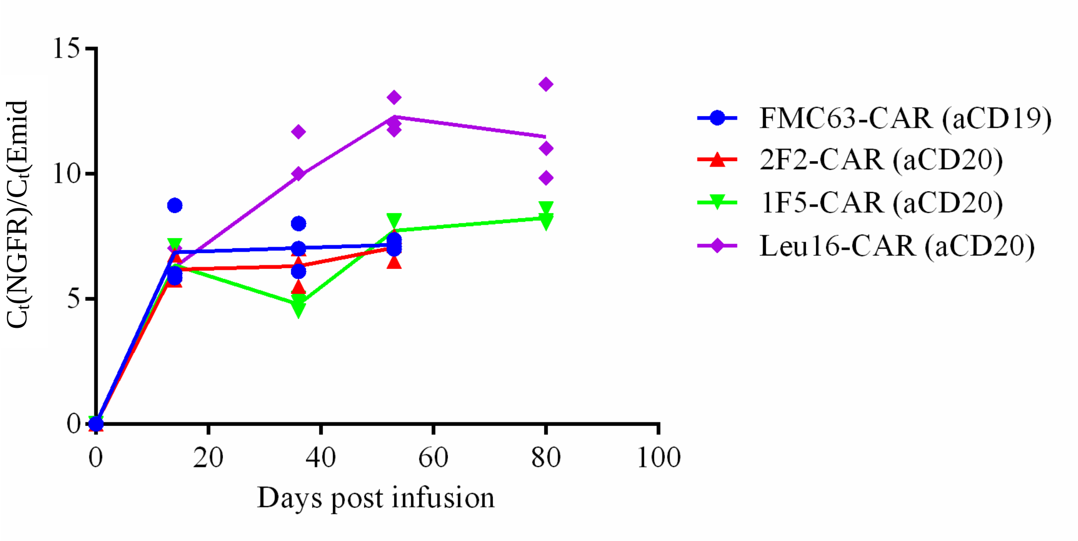

Figure S4. Q-PCR analysis of CAR T cell dynamics in vivo.

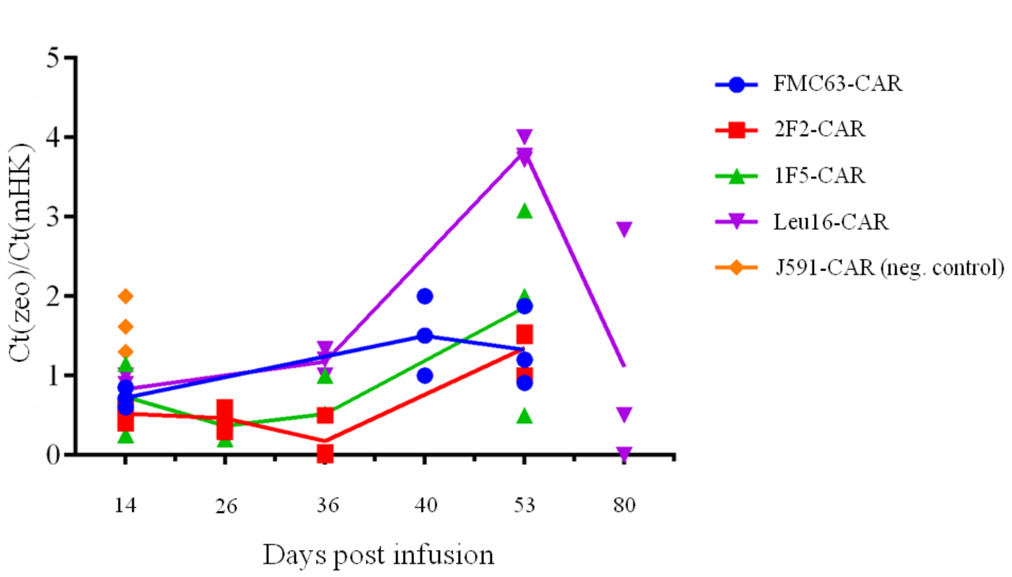

Figure S5. Indirect assessment of tumor cell numbers via qPCR detection of tumor cell DNA in blood samples of Nalm6-CD20 xenotransplanted mice.

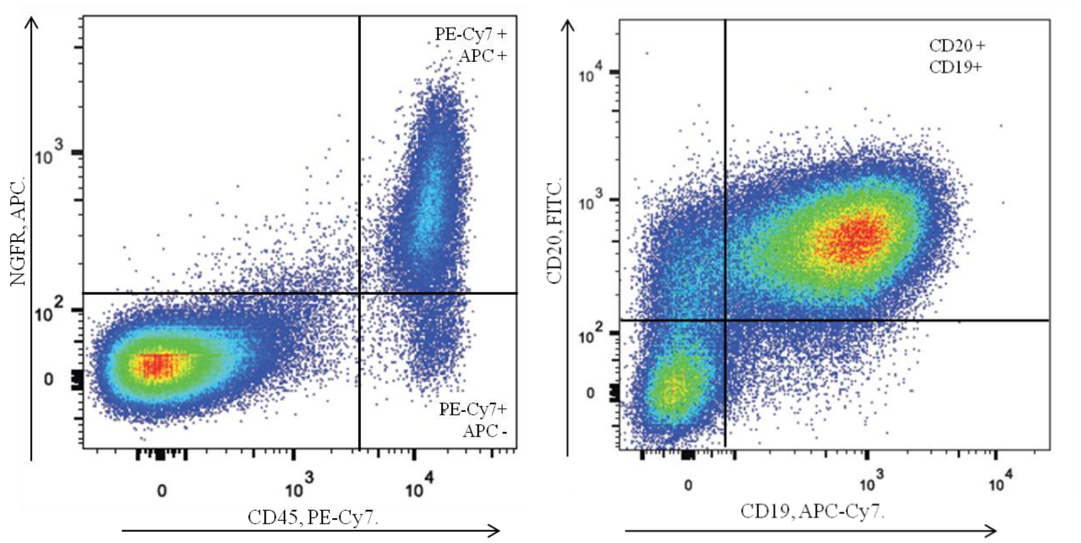

Figure S6. Simultaneous presence of CAR T-cells (left, anti-CD45 and -NGFR staining) and tumor cells (right, anti-CD19 and -CD20) in the bone marrow sample from an euthanized mouse (representative image).
